# Aortic stiffness in aortic stenosis assessed by cardiovascular MRI: a comparison between bicuspid and tricuspid valves

**DOI:** 10.1007/s00330-018-5775-6

**Published:** 2018-11-28

**Authors:** Anvesha Singh, Mark A. Horsfield, Soliana Bekele, John P. Greenwood, Dana K. Dawson, Colin Berry, Kai Hogrefe, Damian J. Kelly, John G. Houston, Prasad Guntur Ramkumar, Akhlaque Uddin, Toru Suzuki, Gerry P. McCann

**Affiliations:** 1 Department of Cardiovascular Sciences, University of Leicester and Cardiovascular Theme, NIHR Leicester Biomedical Research Centre, Glenfield Hospital, Groby road, Leicester, LE3 9QP UK; 2 Xinapse Systems Ltd., West Bergholt, Essex, CO6 3BW UK; 3 Multidisciplinary Cardiovascular Research Centre & The Division of Biomedical Imaging, Leeds Institute of Cardiovascular & Metabolic Medicine, Leeds University, Leeds, LS2 9JT UK; 4 Cardiovascular Medicine Research Unit, School of Medicine and Dentistry, University of Aberdeen, Polwarth Building, Foresterhill, Aberdeen, AB25 2ZD UK; 5 BHF Glasgow Cardiovascular Research Centre, University of Glasgow, 126 University Place, Glasgow, G12 8TA UK; 6 Cardiology Department, Kettering General Hospital Foundation Trust, Rothwell Rd, Kettering, NN16 8UZ UK; 7 Cardiology Department, Royal Derby Hospital, Uttoxeter Rd, Derby, DE22 3NE UK; 8 Division of Molecular & Clinical Medicine, Ninewells Hospital and Medical School, Dundee, DD1 9SY UK

**Keywords:** Aortic valve stenosis, Pulse wave velocity, Aorta, thoracic, Aortic valve, bicuspid, Magnetic resonance imaging

## Abstract

**Objectives:**

To compare aortic size and stiffness parameters on MRI between bicuspid aortic valve (BAV) and tricuspid aortic valve (TAV) patients with aortic stenosis (AS).

**Methods:**

MRI was performed in 174 patients with asymptomatic moderate-severe AS (mean AVAI 0.57 ± 0.14 cm^2^/m^2^) and 23 controls on 3T scanners. Valve morphology was available/analysable in 169 patients: 63 BAV (41 type-I, 22 type-II) and 106 TAV. Aortic cross-sectional areas were measured at the level of the pulmonary artery bifurcation. The ascending and descending aorta (AA, DA) distensibility, and pulse wave velocity (PWV) around the aortic arch were calculated.

**Results:**

The AA and DA areas were lower in the controls, with no difference in DA distensibility or PWV, but slightly lower AA distensibility than in the patient group. With increasing age, there was a decrease in distensibility and an increase in PWV. After correcting for age, the AA maximum cross-sectional area was higher in bicuspid vs. tricuspid patients (12.97 [11.10, 15.59] vs. 10.06 [8.57, 12.04] cm^2^, *p* < 0.001), but there were no significant differences in AA distensibility (*p* = 0.099), DA distensibility (*p* = 0.498) or PWV (*p* = 0.235). Patients with BAV type-II valves demonstrated a significantly higher AA distensibility and lower PWV compared to type-I, despite a trend towards higher AA area.

**Conclusions:**

In patients with significant AS, BAV patients do not have increased aortic stiffness compared to those with TAV despite increased ascending aortic dimensions. Those with type-II BAV have less aortic stiffness despite greater dimensions. These results demonstrate a dissociation between aortic dilatation and stiffness and suggest that altered flow patterns may play a role.

**Key Points:**

• *Both cellular abnormalities secondary to genetic differences and abnormal flow patterns have been implicated in the pathophysiology of aortic dilatation and increased vascular complications associated with bicuspid aortic valves (BAV).*

• *We demonstrate an increased ascending aortic size in patients with BAV and moderate to severe AS compared to TAV and controls, but no difference in aortic stiffness parameters, therefore suggesting a dissociation between dilatation and stiffness.*

• *Sub-group analysis showed greater aortic size but lower stiffness parameters in those with BAV type-II AS compared to BAV type-I.*

**Electronic supplementary material:**

The online version of this article (10.1007/s00330-018-5775-6) contains supplementary material, which is available to authorized users.

## Introduction

Bicuspid aortic valve (BAV) is the most common congenital cardiac anomaly, affecting 1–2% of the general population [1]. It is associated with an increased incidence of aortic root dilatation [2] and vascular complications, with the reported pooled risk of aortic dissection being as high as 4%[3, 4]. Cellular abnormalities, such as cystic medial necrosis and apoptosis, have been observed in the aortic walls of patients with BAV disease [5, 6]. Two dominant hypotheses have been proposed to explain these abnormalities: (i) the changes are due to a primary aortopathy which is genetically mediated [5, 6] and (ii) abnormal flow patterns associated with BAV disease lead to secondary changes in the aorta [7, 8].

The viscoelastic properties of the aorta are essential to maintain proximal and distal arterial flow and organ perfusion, and arterial stiffness has been shown to be independently associated with the development of cardiovascular disease [9]. Pulse wave velocity (PWV), which is the rate of propagation of the systolic wave front down a vessel, is a marker of vessel wall stiffness and inversely related to its distensibility. It can most accurately be measured invasively by intra-arterial pressure measurements; however, its non-invasive assessment is more practical. Magnetic resonance imaging (MRI) allows direct (aortic distensibility) and indirect (PWV) measurement of arterial stiffness in a single examination, at multiple sites, with good agreement compared to invasive measurements and with excellent reproducibility [10]. Abnormalities of aortic stiffness have been found in patients with normally functioning BAVs. Lower aortic distensibility and higher aortic stiffness index on echocardiography or PWV on MRI have been demonstrated in patients with BAV compared to controls [11–14]. The interpretation of these findings was that there is an intrinsic aortopathy associated with BAV.

However, no studies to date have reported on whether there are significant differences in aortic stiffness parameters in patients with aortic stenosis (AS) secondary to BAV disease and degenerative AS of a tricuspid valve (TAV). If patients with BAV did have a genetic predisposition to the development of aortopathy, one would expect stiffness parameters to be increased in comparison to those with TAV. We hypothesised that patients with AS and BAV would have significantly greater thoracic aortic stiffness measured by MRI than patients with tricuspid AS and healthy controls without valve disease.

## Materials and methods

### Study population

Asymptomatic patients with moderate to severe AS were prospectively recruited as part of a multicentre study conducted in the UK (PRIMID-AS study) [15]. Patients with more than mild degree of other valve disease, including aortic regurgitation, were excluded. Asymptomatic controls with no valve disease were also recruited for comparison.

### Ethics, consent, and permissions

The study was approved by the United Kingdom National Research Ethics Service (11/EM/0410) and written informed consent was obtained from all subjects before participation.

### Echocardiography

All subjects underwent a comprehensive trans-thoracic echocardiogram as per international guidelines, to quantify AS severity. In addition, mitral inflow velocities and tissue Doppler imaging (TDI) was used to assess diastolic function. Pulsed-wave Doppler was performed at the mitral valve tips to calculate the E/A ratio, and pulsed-wave TDI was used to measure the septal and lateral mitral annular velocity, for calculation of the septal and lateral E/e’.

### MRI image acquisition

**Fig. 1:**
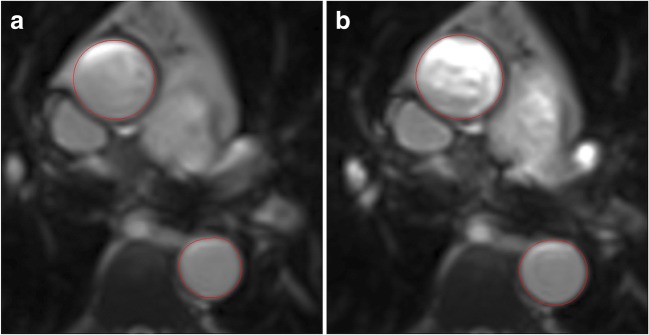
End-diastolic (**a**) and end-systolic (**b**) frames from SSFP cine image of the ascending (top larger region) and descending (bottom smaller region) aorta, used for measurement of aortic dimensions and distensibility calculation

**Fig. 2:**
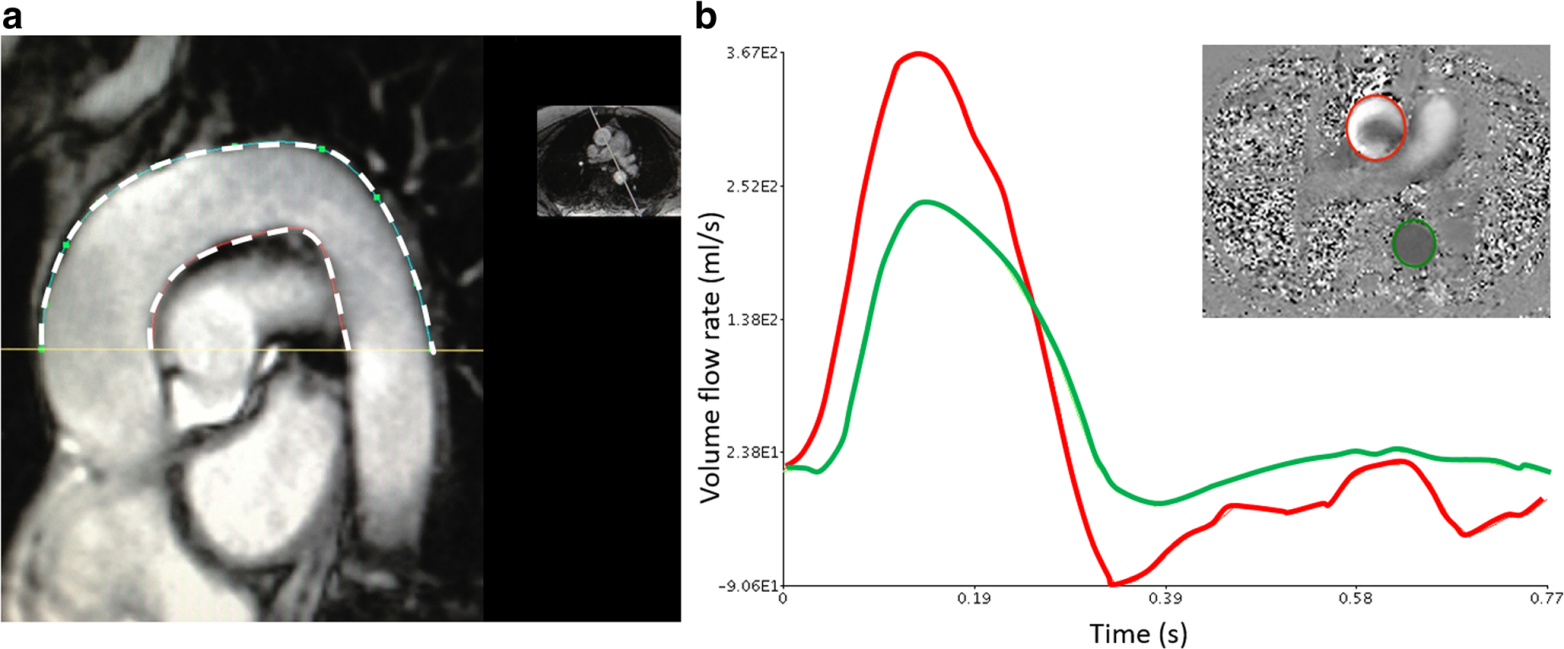
Pulse wave velocity calculation. **a** Sagittal oblique cine of the aorta for measurement of Δ*x* (average of outer and inner distance in white dashed line). **b** Aortic flow sequence used for calculation of Δ*t*, i.e., the transit delay, which was estimated from the cross-correlation between the flow rate curves for the ascending (red) and descending (green) aorta (see text for details)

Cine imaging was performed as previously described [16] on 3T scanners to determine left ventricular (LV) volumes, mass and function. In addition, steady-state free precession (SSFP) cine images of the aortic valve (or gradient-echo if significant artefacts were present), and a high temporal resolution cine image of the ascending (AA) and descending aorta (DA) at the level of the pulmonary artery bifurcation (slice thickness 6 mm, reconstructed to 40 phases, temporal resolution ~ 20 ms, matrix 192 × 256), were acquired. These cine images were used to measure aortic cross-sectional areas throughout the cardiac cycle and calculate distensibility [17] (Fig. 1). A retrospectively gated phase-contrast velocity-encoded sequence (typical parameters: slice thickness 5 mm, VENC 250 cm/s, reconstructed to 100–128 phases, temporal resolution ~ 10 ms, TE 4 ms, matrix 176 × 256), optimised for the study with high temporal resolution and a large number of reconstructed phases, was acquired perpendicular to the ascending and descending thoracic aorta to calculate through-plane flow. As the planning of the slice was far away from the aortic valve, a VENC of 250 cm/s was adequate in most cases, but if aliasing artefact was noted, then a repeat acquisition with a slightly higher VENC was performed. Brachial artery blood pressure was recorded at the time of the aortic cine acquisition to calculate the pulse pressure. A sagittal oblique view of the aortic arch was acquired to calculate the distance between sections for flow measurements in the ascending and descending aorta (Fig. 2). In addition late gadolinium enhancement (LGE) and pre- and post-contrast T1 mapping was performed for calculation of extracellular volume fraction, as previously described [16, 18].

### MRI image analysis

All analyses were performed offline at the core lab, blinded to patient details, by a single observer (AS). LV volume and function were assessed using *cvi42* V5 (circle cardiovascular imaging), excluding papillary muscle and trabeculations from the myocardial mass [19]. Valve morphology was classified according to Schaefer [20] with fusion of left and right coronary cusps in type-I BAV and right and non-coronary cusps in type-II BAV patients (there were no type-III patients in this cohort). Aortic root dimensions were measured at the annulus, sinus of Valsalva, sinotubular junction and proximal AA on the standard 3-chamber and the coronal LVOT views and an average was taken. Ascending and descending maximum and minimum aortic areas were measured from the aortic cine at the pulmonary artery bifurcation level. Aortic distensibility (in 10^−3^ mmHg^−1^) was calculated using the following equation [14, 17]:

$$ \mathrm{Distensibility}=\left[\left(A\max -A\min \right)/\left(A\min \times \mathrm{PP}\right)\right], $$

where *A*max and *A*min are the maximum and minimum aortic cross-sectional areas, and PP is the pulse pressure, i.e., systolic blood pressure − diastolic blood pressure (mmHg).

PWV was assessed in the segment including the ascending aorta, the aortic arch and the proximal descending aorta up to the level of the pulmonary artery bifurcation (Fig. 2). PWV (in m/s) was calculated from:

$$ \mathrm{PWV}=\Delta x/\Delta t, $$

where Δ*x* is the distance around the aortic arch between the two sections through the ascending and descending aorta, and Δ*t* is the transit time delay of two volume flow rate curves for the descending and ascending aorta. The sagittal oblique view of the aorta was used to measure the distance around the aortic arch (Δ*x*), taking the mean of the two distance measures for the outer and inner borders of the aortic lumen. The software package ‘Jim’ (Version 7, Xinapse Systems) was used to calculate Δ*t* from the volume flow rate curves from the AA and DA. A maximum in the cross-correlation between these curves was used to estimate the transit time of the pressure wave around the arch.

### Statistical analysis

Statistical tests were performed using SPSS 24.0 software (Statistical Package for the Social Sciences). Normality was assessed using the Shapiro-Wilk test, histograms and Q-Q plots. For continuous data, summary statistics are expressed as mean ± standard deviation. Log transformation was performed for non-normally distributed data. An independent sample *t* test was used to compare normally distributed or log-transformed data between bicuspid and tricuspid patients, and between patients and controls. The Mann-Whitney test was used for non-parametric data comparison. The bicuspid group was further split by their sub-types (type-I, and type-II) and compared to tricuspid patients, using ANCOVA modelling, adjusted to take age into account. All *p* values < 0.05 were considered statistically significant. Univariate associations of distensibility and PWV were explored using linear regression analysis. Variables were then selected to be entered into a stepwise multivariable model based on statistical significance (*p* < 0.05) or clinical importance, whilst avoiding co-linear variables.

## Results

### Comparison of AS with controls

Of 174 patients recruited, aortic valve morphology classification was possible in 169 (due to missing or unanalysable aortic valve cine imaging in 5 patients), with 63 BAV and 106 TAV patients forming the final population. Distensibility was not measurable in six of these patients due to aortic cine images not being available, and PWV was missing in eight due to missing flow images or artefacts. There was no difference in age and gender distribution between the 23 controls and overall AS group (Table 1). Patients had a higher incidence of hypertension and hyperlipidaemia, although treated hypertension was not excluded from the control group, to assess the incremental effect of AS on LV remodelling. AS patients demonstrated significantly higher LV volumes, mass and mass/volume and more LGE than controls. The aortic root measurements were higher in patients than controls at the annulus and proximal AA levels. The AA and DA areas were higher in patients, with no difference in DA distensibility or PWV, but slightly higher AA distensibility (Table 2).

**Table 1:** Demographic, echocardiographic, and MRI data for those with bicuspid and tricuspid aortic stenosis

Variable	Bicuspid (*n* = 63)	Tricuspid (*n* = 106)	*p* value (bi vs tri)	All AS (*n* = 169)	Controls (*n* = 23)	*p* value (AS vs control)
Age (years)	64.6 [51.1, 69.7]	71.4 [65.8, 77.3]	< 0.001*	69.3 [61.5, 75.6]	66.0 [61.0, 74.8]	0.935
Male (%)	73.0	77.4	0.524	75.7	69.6	0.521
BSA (m^2^)	1.92 ± 0.20	1.97 ± 0.20	0.166	1.95 ± 0.20	1.93 ± 0.18	0.685
Hypertension (%)	44.4	60.4	0.044*	54.4	26.1	0.011*
Hyperlipidaemia (%)	47.6	58.5	0.390	54.4	21.7	< 0.001*
Diabetes (%)	7.9	17.9	0.072	14.2	8.7	0.469
Statin (%)	49.2	67.9	0.016*	60.9	43.5	0.110
ACEI/ARB (%)	42.9	47.2	0.586	45.6	21.7	0.030*
B-blocker (%)	34.9	30.2	0.524	32.0	4.3	0.006*
HR (bpm)	72.1 ± 10.7	69.3 ± 11.6	0.119	70.3 ± 11.3	72.6 ± 8.2	0.364
SBP (mmHg)	142.7 ± 21.3	149.3 ± 20.6	0.050	146.8 ± 21.1	154.1 ± 25.0	0.132
DBP (mmHg)	80.6 ± 11.0	74.9 ± 9.9	0.001*	77.0 ± 10.6	82.0 ± 9.4	0.032*
PP (mmHg)	62.01 ± 16.4	74.41 ± 19.9	< 0.001*	69.8 ± 19.5	72.02 ± 24.0	0.621
Echocardiographic data						
AV Vmax (m/s)	3.96 ± 0.56	3.79 ± 0.55	0.057	3.86 ± 0.56	1.36 ± 0.27	< 0.001*
Mean gradient (mmHg)	37.9 ± 13.1	33.7 ± 11.6	0.032*	35.3 ± 12.3	4.2 ± 1.6	< 0.001*
AVAI (cm^2^/m^2^)	0.58 ± 0.16	0.57 ± 0.13	0.773	0.57 ± 0.14	1.71 ± 0.36	< 0.001*
E/A	0.92 ± 0.32	0.85 ± 0.27	0.204	0.88 ± 0.29	0.84 ± 0.23	0.523
Septal E/e’	12.14 ± 4.62	12.44 ± 5.02	0.734	12.33 ± 4.86	10.67 ± 3.34	0.115
Lateral E/e’	9.24 ± 3.96	10.21 ± 3.63	0.073	9.86 ± 3.77	8.07 ± 2.97	0.031*
MRI data						
LVEDVI (ml/m^2^)	88.3 ± 18.4	86.4 ± 18.1	0.505	87.1 ± 18.2	78.2 ± 9.4	0.001*
LVEF (%)	57.0 ± 4.1	56.7 ± 5.1	0.668	56.8 ± 4.8	58.9 ± 3.7	0.050
LVMI (g/m^2^)	56.5 [47.2, 66.4]	54.9 [47.7, 64.5]	0.649	55.6 [47.6, 65.6]	42.2 [39.7, 47.9]	< 0.001*
LV mass/volume	0.66 ± 0.12	0.67 ± 0.11	0.631	0.66 ± 0.11	0.57 ± 0.08	< 0.001*
LGE (g)	2.67 [0.77, 4.96]	2.20 [0.83, 6.22]	0.904	2.44 [0.81, 6.08]	0.77 [0.40, 2.22]	0.001*
ECV (%)	24.48 ± 2.46	24.91 ± 2.33	0.350	24.8 ± 2.4	25.05 ± 2.57	0.590
Annulus (mm)	25.53 ± 2.69	23.76 ± 2.44	< 0.001*	24.42 ± 2.67	22.82 ± 2.23	0.006*
SoV (mm)	34.82 ± 4.00	32.92 ± 3.81	0.002*	33.63 ± 3.98	33.50 ± 3.78	0.880
STJ (mm)	29.52 ± 3.88	27.16 ± 3.67	< 0.001*	28.04 ± 3.91	27.41 ± 3.00	0.456
Proximal AA (mm)	36.40 ± 5.22	31.42 ± 4.13	< 0.001*	33.27 ± 5.15	29.75 ± 2.93	< 0.001*

Data presented as mean, with standard deviation in parentheses or median with 25th and 75th centile in parenthesis

*SBP* systolic blood pressure, *DBP* diastolic blood pressure, *AVAI* aortic valve area indexed to body surface area, *MRI* magnetic resonance imaging, *LVEDVI* left ventricular end-diastolic volume index, *LVMI* left ventricular mass index, *LVEF* left ventricular ejection fraction, *SoV* sinus of valsalva, *STJ* sinotubular junction, *AA* ascending aorta

*Significant difference on unpaired *t* test or Mann Whitney-U test, as appropriate

**Table 2:** Aortic area, distensibility, and pulse wave velocity in bicuspid and tricuspid groups

	Bicuspid (*n* = 63)	Tricuspid (*n* = 106)	*p* value (bi vs tri)	*p* value after correcting for age (bi vs tri)	Controls (*n* = 23)
AAmax (cm^2^)	12.97 [11.10, 15.59]	10.06 [8.57, 12.04]	< 0.001*	< 0.001*	9.46 [7.85, 10.48] ^**†**^
DAmax (cm^2^)	5.84 [4.76, 6.59]	6.07 [5.17, 7.32]	0.020*	0.902	5.18 [4.68, 5.92] ^**†**^
AAmin (cm^2^)	11.57 [10.08, 14.11]	9.00 [7.56, 10.66]	< 0.001*	< 0.001*	8.98 [7.24, 9.49] ^**†**^
DA min (cm^2^)	5.27 [4.07, 5.74]	5.52 [4.67, 6.66]	0.013*	0.980	4.54 [4.27, 5.53] ^**†**^
AA distensibility (10^-3^ mmHg^-1^)	1.64 [1.17, 3.02]	1.58 [1.20, 2.07]	0.396	0.099	1.23 [0.73, 1.73] ^**†**^
DA distensibility (10^-3^ mmHg^-1^)	2.45 [1.43, 2.89]	1.55 [1.10, 2.18]	< 0.001*	0.498	1.40 [1.05, 1.95]
PWV (m/s)	7.44 [4.88, 10.51]	7.88 [6.32, 9.92]	0.150	0.235	8.20 [6.83, 8.97]

Data presented as median [25th, 75th percentile]. Column-4 shows *p* values using the Mann-Whitney *U* test; column-5 shows *p* values after correcting for age, using one-way ANCOVA test of log-transformed data *p < 0.05

*AAmax/min* maximum/minimum ascending aortic area, *DAmax/min* maximum/ minimum descending aortic area, *PWV* pulse wave velocity

^†^*p* < 0.05 for comparison of controls with AS patients after correcting for age using one-way ANCOVA test of log-transformed data

### Comparison between bicuspid and tricuspid groups

#### Baseline characteristics

The BAV group was significantly younger, with a lower incidence of hypertension and statin use and higher diastolic blood pressure than those with tricuspid AS (Table 1). The TAV group also had higher mean pressure gradient but similar peak gradient and aortic valve area index. Both groups had similar degree of LV remodelling, late gadolinium enhancement and extracellular volume fraction. The aortic root diameters were larger in BAV than TAV group at all levels (Table 1).

#### Aortic area, distensibility and PWV

**Fig. 3:**
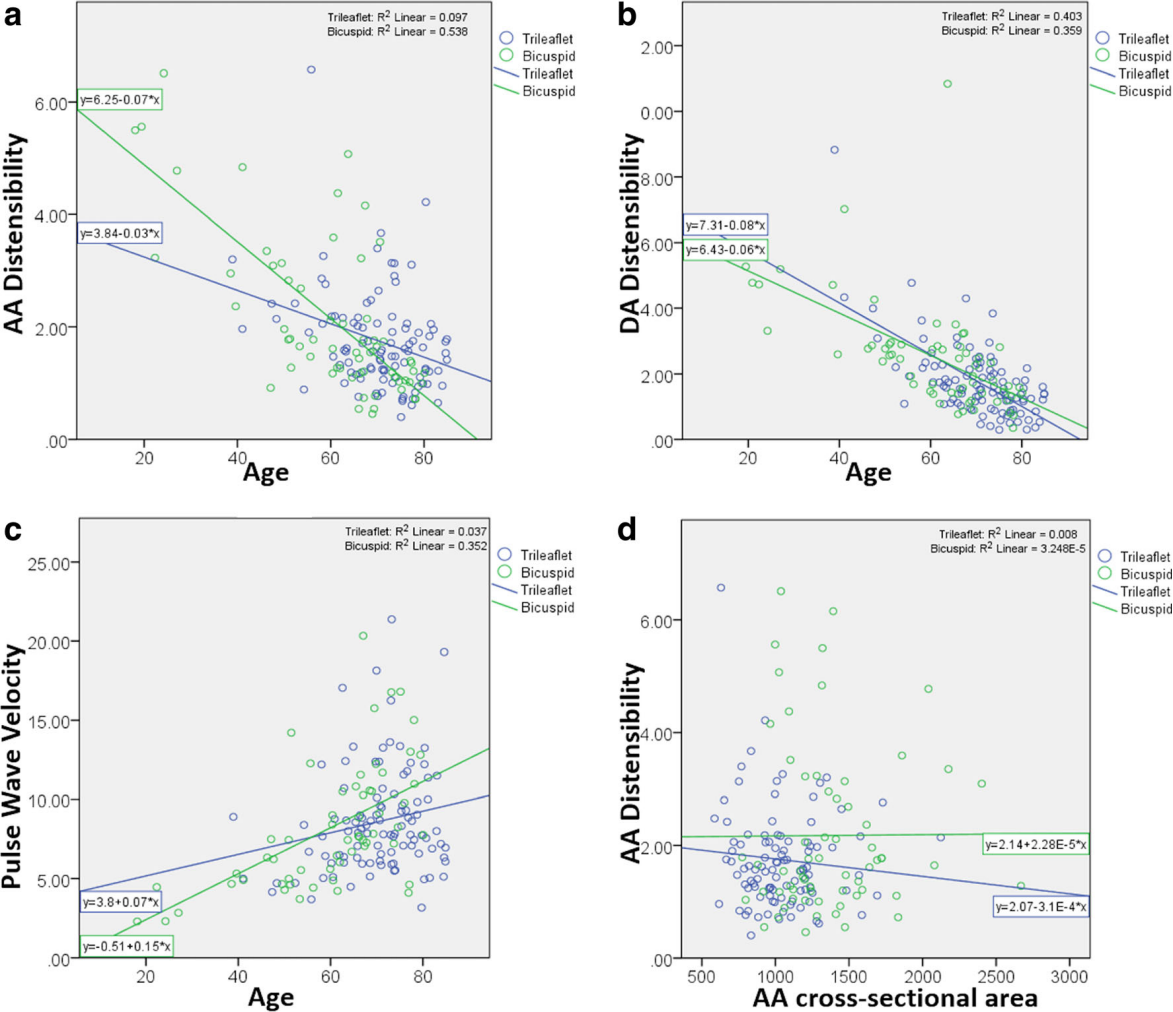
Scatter plots showing the relationship of age with distensibility (**a**, **b**) and pulse wave velocity (**c**) and of distensibility with cross-sectional area in the ascending aorta (**d**). Units—distensibility, 10^−3^ mmHg^−1^; PWV, m/s; AA area, mm^2^; age years

The maximum and minimum cross-sectional AA areas were significantly higher in bicuspid patients and this remained statistically so even after correcting for age (Table 2). There was no difference in the age-corrected DA cross-sectional area. With increasing age, there was a decrease in distensibility (*r* = -0.45, *p* < 0.001 for the AA; *r* = -0.64, *p* < 0.001 for DA) and an increase in PWV (*r* = 0.38, *p* < 0.001) (Fig. 3a–c). After correcting for age, there was no significant difference in AA distensibility, DA distensibility or PWV between bicuspid and tricuspid patients (Table 2). In addition, AA area did not correlate with AA distensibility (Fig. 3d).

#### Comparison by bicuspid type-I, and type-II sub-groups

**Fig. 4:**
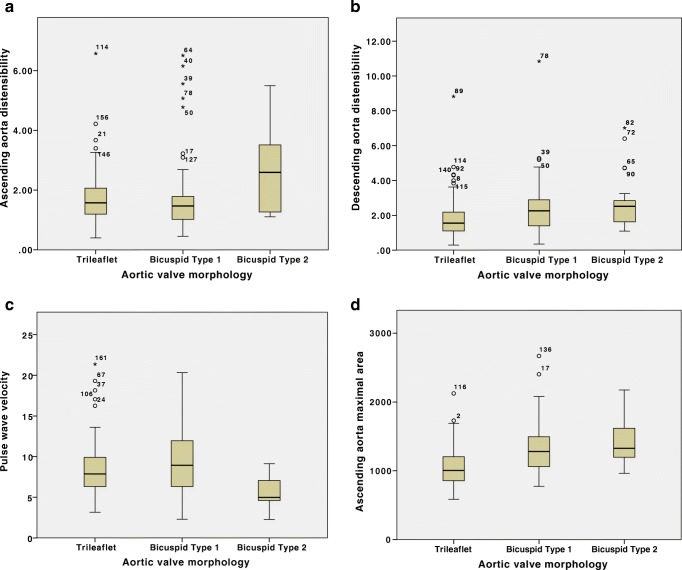
Box plots showing the ascending aorta distensibility (**a**), descending aorta distensibility (**b**), pulse wave velocity (**c**), and maximum ascending aorta area (**d**) for patients with tricuspid, bicuspid type-I, and bicuspid type-II aortic valves. Units as in Fig. 3

There were no significant differences in age, gender, resting haemodynamics, LV function or AS severity between the two BAV sub-groups. There were significant differences in age-corrected AA area, AA distensibility and PWV between the three groups (tricuspid, bicuspid type-I, and type-II) (Table 3 and Fig. 4). Post-HOC analysis revealed a significantly higher AA area in both bicuspid sub-groups compared to tricuspid patients, and a significantly higher AA distensibility and lower PWV in the bicuspid type-II sub-group compared to bicuspid type-I patients. There were no significant differences in the DA area or distensibility between the sub-groups.

**Table 3:** Aortic area, distensibility, and pulse wave velocity in tricuspid, bicuspid type-I, and type-II sub-group comparison using age as a covariate

	Tricuspid (*n* = 106)	Bicuspid type-I (*n* = 41)	Bicuspid type-II (*n* = 22)	*p* value (AS sub-groups only)	Controls (*n* = 23)	*p* value (AS sub-groups and controls)
AA max (cm^2^)	10.06 [8.57,12.04]^**† ‡**^	12.82 [10.62,14.95]	13.28 [11.97, 16.20]	< 0.001*	9.46 [7.85, 10.48]^**† ‡**^	< 0.001*
DA max (cm^2^)	6.07 [5.17, 7.32]	5.89 [4.87, 6.60]	5.32 [4.05, 6.54]	0.953	5.18 [4.68, 5.92]	0.102
AA distensibility (10^−3^ mmHg^−1^)	1.58 [1.20, 2.07]^**†**^	1.47 [1.02, 1.79]^**‡**^	2.60 [1.27, 3.51]	0.010*	1.23 [0.73, 1.73]**°**^**‡**^	< 0.001*
DA distensibility (10^−3^ mmHg^−1^)	1.55 [1.10, 2.18]	2.26 [1.40, 2.89]	2.51 [1.63, 2.84]	0.729	1.40 [1.05, 1.95]	0.638
PWV (m/s)	7.88 [6.32, 9.92]^**†**^	8.94 [6.34, 11.96]^**‡**^	4.99 [4.61, 7.06]	0.001*	8.20 [6.83, 8.97]	0.002*

Abbreviations same as for Table 2. Data presented as median [25th, 75th centile]. Age-corrected *p* values obtained using ANCOVA test of log-transformed values with age as a covariate

*p < 0.05

^†^Significant difference compared to bicuspid type-I

^**‡**^Significant difference compared to bicuspid type-II

**°**Significant difference compared to tricuspid on post-HOC analysis using Bonferroni test

### Associations of AA distensibility and PWV

The univariate associations of AA distensibility and PWV in the patient group, both before and after adjusting for age, are summarised in supplemental Tables 1 and 2. Age was the only independent factor associated with both parameters after entering the following variables into a stepwise multivariable model: age, sex, BMI, valve subtype, diabetes, hypertension (or PP instead for PWV), and eGFR.

## Discussion

Our results show no statistically significant differences in aortic distensibility and PWV between bicuspid and tricuspid patients with AS, despite increased AA diameters in those with BAV. On further analysis, we found bicuspid type-II patients to have higher AA distensibility and lower aortic stiffness (PWV) than their type-I counterparts, despite a trend towards the highest AA area. The strengths of this study are the multicentre design, core lab blinded analysis, moderately large population, classification of valve morphology, and measurement of aortic stiffness with MRI, which allowed localised assessment in the area of interest.

### Aortic area

We have confirmed findings from previous echocardiographic studies that patients with BAV have a greater degree of AA dilatation compared to tricuspid controls, either with or without valvular stenosis or regurgitation [21], and out of proportion to the degree of valvular dysfunction [2, 22]. Several studies have also looked at the relationship between the morphology of BAV and aortic dimensions, with mixed findings. Novaro et al found type-II BAV patients to have slightly higher mid-ascending aortic dimensions compared to type-I, though this did not reach statistical significance [23]. Type-II BAVs have also been associated with a larger aortic arch and ascending aorta [20]. However, Cecconi et al found no difference in aortic dimensions in 162 younger patients with type-1 and type-II BAV, although the average age of that cohort was only 23.6 years and aortic dimensions did strongly correlate with age [24].

### Aortic stiffness

Distensibility and PWV are markers of arterial wall stiffness that are inversely related according to the Bramwell and Hill equation [25] and reflect the elastic properties of the aorta. Aortic distensibility is principally governed by the composition of the aortic wall intra-cellular matrix and luminal mean arterial pressure. Previous MRI studies have also shown that distensibility decreases and PWV increases with age [17, 26, 27], which is the likely reason for the stiffness parameters in our controls, who were older compared to the BAV group. Echocardiographic and MRI studies have found lower distensibility and higher aortic stiffness parameters in BAV patients without significant stenosis or regurgitation, compared to controls [11, 13]. In one echocardiographic study of 32 BAV patients with AS and 32 controls, aortic stiffness index was higher in the BAV group, but there was no significant difference in distensibility between the groups [12]. This suggests that the presence of stenosis may result in progressively reduced distensibility and increased PWV in both bicuspid and tricuspid patients, leading to no significant difference between the two groups. Support for this also comes from marked improvement in aortic stiffness in patients with severe AS 1 year after aortic valve replacement [28].

### Pathophysiology of aortic dilatation

The exact mechanism leading to dilatation of the aorta in bicuspid patients is unclear. Histopathological studies have demonstrated changes in the AA walls of bicuspid patients including cystic medial necrosis [6]. High rates of apoptosis in the aortic media of bicuspid patients both with and without dilatation have been shown, suggesting that apoptosis is a key mechanism for smooth muscle cell loss in the ascending aortas of bicuspid patients, and supporting the hypothesis of a developmental fault involving the valve and aortic wall [5]. However, a similarly high rate of apoptosis was also found in tricuspid patients with aortic dilatation, suggesting a role of extrinsic forces on the aortic wall, rather than an intrinsic developmental abnormality alone. Cystic medial necrosis can be found in hereditary connective tissue disorders such as Marfan syndrome, which has consistently been associated with increased aortic stiffness [29, 30], but a similar histological picture can also be caused by infection, atherosclerosis or severe shear stress [5].

We have shown a dissociation between aortic dilatation and stiffness in BAV disease, with type-II group having lower stiffness parameters, despite a trend towards a higher AA area, further confounding the theory of intrinsic aortic wall stiffness alone leading to aortic dilatation in bicuspid patients. In fact, there was no correlation between AA distensibility and AA dimensions (Fig. 3d). This may suggest a more central role of asymmetrical flow patterns and worse turbulence [31] that has been demonstrated in BAV compared to TAV [7, 8], which also correlated with the degree of proximal aortic dilatation [32]. Recently, time-resolved three-dimensional phase-contrast MRI, also called 4D flow, has demonstrated right-handed helical flow and right-anterior flow jets in type-I BAV, and left-handed helical flow with left-posterior flow jets in type-II BAV [31].

In abdominal aortic aneurysms (AAA), Wilson et al demonstrated increasing aortic distensibility to be an independent predictor of rupture and suggested that this may be due to failure of aortic wall remodelling, which leads to further dilatation and risk of rupture [33]. This is supported by another study showing no correlation between AAA distensibility and size [34]. The authors speculated that the wall of the rapidly expanding AAA may lose its integrity leading to a paradoxically increased distensibility, which may also in part explain our findings in type-II BAV patients.

There appear to be two key players: age being a key determinant of aortic stiffness, with valve haemodynamics (morphology ± presence of stenosis/regurgitation) being central to aortic dilatation. In a previously published set of young controls using the same methodology, the median AA distensibility was 6.36 × 10^−3^ mmHg^−1^ and PWV was 3.97 m/s [35], demonstrating much lower stiffness than this group of patients or older controls. Cecconi found no difference in aortic size between young BAV type-I, and type-II patients with no stenosis/regurgitation [24], whereas type-II BAV patients with mixed valve disease (dysfunctional valves) had larger aortas but similar distensibility to type-I patients in Schaefer’s study [36]. Finally, our older cohort of BAV and TAV with AS demonstrated no significant difference in aortic stiffness, despite larger aortas in BAV.

## Limitations

The cross-sectional nature of the observations precludes any inference about the causality of the associated observations, and the results should be considered hypothesis generating. The bicuspid group were younger, and age is an important determinant of aortic stiffness. However, BAV presents at an earlier age, and we corrected for age in our analysis to try and overcome this limitation. We cannot exclude the possibility that the omission from the study of younger patients with BAV and complications, such as aortic dissection, potentially biased the results by excluding those at highest risk. Sub-group analysis of BAV was limited with relatively small sample size. Four-dimensional flow sequences were not acquired and therefore we could not correlate flow pattern with measures of stiffness. We also did not correct for longitudinal motion of the aortic root during systole, though the measurement was made more distally in the ascending aorta.

## Conclusions

In patients with significant AS, BAV patients do not have increased aortic stiffness compared to those with TAV despite increased ascending aortic dimensions. The AA of patients with type-II BAV has the highest distensibility and lowest PWV despite the greatest dimensions. These results demonstrate a dissociation between aortic dilatation and stiffness and suggest that altered flow patterns may play a role which requires further assessment with longitudinal studies.

## Electronic supplementary material

ESM 1 (DOCX 19.5 kb)

## Abbreviations

AA: Ascending aorta
AS: Aortic stenosis
BAV: Bicuspid aortic valve
DA: Descending aorta
LGE: Late gadolinium enhancement
LV: Left ventricular
MRI: Magnetic resonance imaging
PP: Pulse pressure
PWV: Pulse wave velocity
TAV: Tricuspid aortic valve
