# Supplementary material for: Aortic stiffness in aortic stenosis assessed by cardiovascular MRI: a comparison between bicuspid and tricuspid valves
Source: Eur Radiol. 2018 Nov 28;29(5):2340–9. doi: 10.1007/s00330-018-5775-6 (PMC6443917; doi:10.1007/s00330-018-5775-6)
Supplement: Supplementary file 1 — (DOCX 19.5 kb) [file 330_2018_5775_MOESM1_ESM.docx]

# Supplemental material

**Supplemental Table 1.** Univariate associations with PWV

| **Variable** | **Estimate (95% CI)** | **p-value** | **p-value after adjusting for age** |
| --- | --- | --- | --- |
| **Age** | 0.10 (0.07, 0.14) | **<0.001** | N/A |
| **Sex (M)** | 1.52 (0.26, 2.78) | **0.019** | 0.066 |
| **BSA** | -0.68 (-3.38, 2.02) | 0.620 | 0.792 |

| **BMI** | -0.09 (-0.22, 0.05) | 0.203 | 0.164 |
| --- | --- | --- | --- |

| **HR** | -0.02 (-0.07, 0.03) | 0.437 | 0.941 |
| --- | --- | --- | --- |
| **SBP** | 0.03 (0.00, 0.06) | **0.022** | 0.477 |
| **DBP** | 0.02 (-0.03, 0.07) | 0.374 | 0.134 |
| **PP** | 0.03 (0.00, 0.06) | **0.047** | 0.925 |
| **Log(NTproBNP)** | 0.19 (-0.11, 0.49) | 0.207 | 0.240 |
| **eGFR** | -0.03 (-0.05, -0.01) | **0.002** | 0.946 |
| **Hba1c** | 0.37 (-0.38, 1.12) | 0.328 | 0.858 |
| **Diabetes** | 0.01 (-1.55, 1.57) | 0.991 | 0.767 |
| **Hypertension** | 0.84 (-0.25, 1.94) | 0.130 | 0.963 |
| **AoV subtype (bi vs tri)** | 0.52 (-0.64, 1.67) | 0.377 | 0.168 |
| **AoV subtype (3 subgroups)** | 3.71 (1.89, 5.54) | **<0.001** | **0.001** |
| **AV Vmax** | -0.18 (-1.15, 0.80) | 0.720 | 0.412 |
| **MPG** | -0.01 (-0.05, 0.03) | 0.677 | 0.391 |
| **AVAI** | 0.64 (-3.33, 4.61) | 0.750 | 0.164 |
| **VAI (CMR)** | 0.29 (-0.37, 0.95) | 0.391 | 0.202 |
| **LVMI** | 0.01 (-0.03, 0.05) | 0.519 | 0.167 |
| **LV mass/vol** | -0.60 (-5.52, 4.32) | 0.810 | 0.449 |
| **LGE (g)** | 0.07 (-0.05, 0.19) | 0.240 | 0.860 |
| **Native T1** | 0.01 (-0.00, 0.02) | 0.133 | 0.181 |
| **ECV** | 0.09 (-0.20, 0.38) | 0.523 | 0.917 |

**Supplemental Table 2.** Univariate associations with AA Distensibility

| **Variable** | **Estimate (95% CI)** | **p-value** | **p-value after adjusting for age** |
| --- | --- | --- | --- |
| **Age** | -0.05 (-0.06, -0.04) | **<0.001** | N/A |
| **Sex (M)** | -0.36 (-0.79, 0.06) | 0.090 | 0.491 |
| **BMI** | -0.01 (-0.05, 0.04) | 0.791 | 0.923 |
| **HR** | 0.01 (-0.00, 0.03) | 0.094 | 0.671 |
| **SBP** | -0.02 (-0.03, -0.02) | **<0.001** | **<0.001** |
| **DBP** | 0.00 (-0.02, 0.02) | 0.841 | 0.712 |
| **PP** | -0.03 (-0.04, -0.02) | **<0.001** | **<0.001** |
| **Log(NTproBNP)** | -0.15 (-0.25, -0.05) | **0.002** | 0.528 |
| **eGFR** | 0.01 (0.01, 0.02) | **<0.001** | 0.138 |
| **Hba1c** | -0.25 (-0.52, 0.03) | 0.079 | 0.708 |
| **Diabetes** | -0.00 (-0.52, 0.51) | 0.992 | 0.674 |
| **Hypertension** | -0.54 (-0.90, -0.19) | **0.003** | 0.383 |
| **AoV subtype (bi vs tri)** | -0.43 (-0.80, -0.06) | **0.023** | 0.328 |
| **AoV subtype (3 subgroups)** | -0.72 (-1.32, -0.12) | **0.005** | 0.115 |
| **AV Vmax** | 0.01 (-0.32, 0.34) | 0.954 | 0.414 |
| **MPG** | -0.00 (-0.02, 0.01) | 0.757 | 0.747 |
| **AVAI** | 0.43 (-0.89, 1.74) | 0.523 | 0.484 |
| **VAI (CMR)** | -0.38 (-0.60, -0.17) | **0.001** | 0.581 |
| **LVMI** | 0.00 (-0.01, 0.02) | 0.765 | 0.548 |
| **LV mass/vol** | -0.53 (-2.15, 1.09) | 0.521 | 0.876 |
| **LGE (g)** | -0.03 (-0.07, 0.01) | 0.183 | 0.535 |
| **LGE %** | -0.04 (-0.09, 0.01) | 0.110 | 0.422 |
| **Native T1** | -0.00 (-0.01, 0.00) | 0.279 | 0.345 |
| **ECV** | 0.02 (-0.08, 0.12) | 0.742 | 0.108 |
